# The Morphology of Hydroxyapatite Nanoparticles Regulates Cargo Recognition in Clathrin-Mediated Endocytosis

**DOI:** 10.3389/fmolb.2021.627015

**Published:** 2021-03-04

**Authors:** Cheng Zhu, Xuejie Zhou, Ziteng Liu, Hongwei Chen, Hongfeng Wu, Xiao Yang, Xiangdong Zhu, Jing Ma, Hao Dong

**Affiliations:** ^1^ Kuang Yaming Honors School, Nanjing University, Nanjing, China; ^2^ Tianjin Key Laboratory of Function and Application of Biological Macromolecular Structures, School of Life Sciences, Tianjin University, Tianjin, China; ^3^ Key Laboratory of Mesoscopic Chemistry of Ministry of Education, Institute of Theoretical and Computational Chemistry, School of Chemistry and Chemical Engineering, Nanjing University, Nanjing, China; ^4^ National Engineering Research Center for Biomaterials, Sichuan University, Chengdu, China; ^5^ Nanxin Pharm. Co., Ltd., Nanjing, China; ^6^ Institute for Brain Sciences, Nanjing University, Nanjing, China

**Keywords:** nanoparticles, conformational change, endocytosis, cancer, molecular modeling and simulation, adaptin

## Abstract

The clathrin-associated protein adaptin-2 (AP2) is a distinctive member of the hetero-tetrameric clathrin adaptor complex family. It plays a crucial role in many intracellular vesicle transport pathways. The hydroxyapatite (HAp) nanoparticles can enter cells through clathrin-dependent endocytosis, induce apoptosis, and ultimately inhibit tumor metastasis. Exploring the micro process of the binding of AP2 and HAp is of great significance for understanding the molecular mechanism of HAp’s anti-cancer ability. In this work, we used molecular modeling to study the binding of spherical, rod-shaped, and needle-shaped HAps toward AP2 protein at the atomic level and found that different nanoparticles’ morphology can determine their binding specificity through electrostatic interactions. Our results show that globular HAp significantly changes AP2 protein conformation, while needle-shaped HAP has more substantial binding energy with AP2. Therefore, this work offers a microscopic picture for cargo recognition in clathrin-mediated endocytosis, clarifies the design principles and possible mechanisms of high-efficiency nano-biomaterials, and provides a basis for their potential anti-tumor therapeutic effects.

## Introduction

The bioactivity and biocompatibility of nanomaterials, notably their antitumor therapeutic effects, have been the focus of recent medical investigations. Various models systems of nanoparticles have been evaluated for their clinical potentials and applied to cancer cells (Brigger et al., 2012). The hydroxyapatite (HAp) nanoparticles could enter cells through the clathrin-dependent endocytosis process, stimulating mitochondria-dependent apoptosis and eventually suppressing tumor proliferation (Chu et al., 2012). Nanosheets of metal dichalcogenides (WS_2_ and MoS_2_) were also internalized by epithelial and macrophage cells, colocalized with lysosomes, and induced ferroptotic cell death in the mouse lung tissues (Hao et al., 2017). Calcium peroxide (CaO_2_) nanoparticles exhibited dual functions of calcium overload and oxidative stress under tumor microenvironment, inhibiting the tumor growth *in vivo* (Zhang et al., 2019b). Hence, the ability to understand and manipulate the interplay between nanoparticles and the cellular environment is vital to innovative ways of developing nano-medicines.

Despite the recent advancements in applying nanoparticles against tumor growth or metastasis, the underlying molecular mechanisms, specifically the interactions between nanoparticles and endocytosis systems (clathrin, adaptor proteins, membrane receptors, etc.) have not been fully explored. The key factors that affect nanoparticles’ functions as biomedicines, such as particle size, morphology, chemical compositions, etc., remain largely elusive. Nanoparticles enter the cells by endocytosis, and cells regulated the movements of extracellular molecules (including nanomaterials) with coated vesicular carriers. Specifically, clathrin-coated vesicles mediate multiple trafficking routes, including internalization from the plasma membrane (Kovtun et al., 2020). Among the clathrin-associated proteins, adaptin-2 (AP2) is the most abundant endocytic clathrin adaptor and a functional hub linking the cargo molecules and the clathrin cage. AP2 consists of four subunits (α, β2, μ2, and σ2), (Jackson et al., 2010) which can adopt a range of conformations under different conditions. To interact with cargos, the AP2-μ2 subunit undergoes a conformational change from a “locked” state to an “open” state, exposing the YxxΦ motif in the C-terminal region and facilitating recruitment of proteins, lipids or nanoparticles (Traub and Bonifacino, 2013).

We previously characterized the HAp nanoparticles and their inhibitory activities against tumor cells or promoting activities on normal tissue cells, and elucidated the downstream signaling pathways evoked by HAp-internalization and others (Chen et al., 2013; Zhao et al., 2017; Wang et al., 2018a; Zhang et al., 2019a; Liu et al., 2019; Wang et al., 2019; Wu et al., 2019; Li et al., 2020). If the cells were pretreated with chlorpromazine (clathrin-pathway inhibitor) (Shi et al., 2017) or NaN_3_ (ATP inhibitor), (Ryan et al., 2015) the uptake of HAp were significantly prohibited, indicating clathrin-mediated and ATP-dependent endocytosis for HAp nanoparticles. The physicochemical properties of HAp as well as its interactions with proteins (such as collagen) or substrates have also been extensively studied at atomic level (Cheng et al., 2017; Wang et al., 2018b; Wang et al., 2018c; Liu et al., 2018; Xie et al., 2018; Gu et al., 2019; Xue et al., 2019; Liu et al., 2020; Ma et al., 2020; Tan et al., 2020; Wang et al., 2020). However, only a few studies have reported the clathrin-mediated adhesion and endocytosis of HAp, (Shi et al., 2017; Shi et al., 2018; Huang et al., 2020) while the molecular details remain elusive. Modeling the dynamic process of HAp binding with AP2 at the atomic level is crucial for a detailed understanding of the driving forces, especially the early events at the nano-biological interface. In the current study, we investigated the interplay of HAp with the upstream pathway of endocytosis to uncover the main factors governing the HAp-AP2 interactions, and to illuminate the design principle of highly efficient nano-biomaterials.

### Computational Details

Construction of nanoparticle structural models. The models of hydroxyapatite (sphere, rod, and needle morphology) were built by Materials Studio (Accelrys, 2006). All the structures were optimized using DFT methods at the level of PBE/DND4.4 in the DMol^3^ package. SwissParam (http://www.swissparam.ch/) was used to generate the CHARMM force field parameters and topology files for each nanoparticle (Zoete et al., 2011). The Mulliken charge was used to generate the electrostatic potential surface. As shown later, all the HAp structures could be well maintained in the following molecular dynamics (MD) simulations, and therefore validates that the parameters can well describe the HAps.

Molecular Dynamics Simulations. The all-atom MD simulations were performed on Gromacs 5.1.1 (Pronk et al., 2013) with CHARMM27 force field (Best et al., 2012) and explicit solvent model TIP3P for water (Jorgensen et al., 1983). The crystal structure of AP2-μ2 subunit (PDB entry: 2XA7. pdb) (Jackson et al., 2010) was adopted as the initial structural model. The missing residues, Q136‐Q141 and K224-K235 (both are loops), were built by MODELLER (Fiser et al., 2000).

The initial binding conformations of AP2-HAp complexes were constructed on HADDOCK2.2.(van Zundert et al., 2016). For the initial poses, we also considered the following criteria: 1) biological-relevance. The AP2-μ2 domain was known to mediate cargo binding, while α, β2 domains mostly register μ2 domain in place or interact with the plasma membrane. Within the μ2 domain, the C-terminal region undergoes a significant conformational change upon ligand binding, hence, the C-terminal region is likely enriched with ligand-interacting sites; and 2) compatible with electrostatic potential. As shown in Supplementary Figure S3, docking on the full-length AP2 protein or the AP2-μ2 show consistent results. Then, we performed MD simulations using the aforementioned three initial poses, and only the site one gives meaningful results, as described later.

The system was then solvated, neutralized with 150 mM NaCl, minimized using steepest descent method, and then equilibrated for 1 ns at 300 K. Simulated annealing was applied to accelerate the sampling and a typical annealing procedure was: starting from 300 K, the system was heated to 500 K within 5 ns, and then gradually cooled to 300 K within 40 ns, and then to 200 K within another 80 ns. After obtaining a stable configuration of the nanoparticle, MD simulations were carried out to accumulate another 100 ns trajectory. Throughout the simulations, velocity-rescale thermostat and constant pressure (1 bar, Parrinello-Rahman NPT ensemble) were adopted. The nonbonded interaction cut-off for electrostatics calculations was set as 10 Å and the particle mesh Ewald (PME) method was used in the calculation of long-range electrostatic interactions. For each system, two independent simulations were carried out to improve the statistic.

For systems with some residues mutated, we started from the aforementioned systems that have been well equilibrated. Another 10 ns MD simulations were carried out after mutation, and 200 frames were evenly extracted from the trajectory to do the following binding free energy calculations with MM/PBSA.

The principal component calculations**.** To detect possible transition between the open and the locked configurations of the AP2 μ2 domain, we calculated a principal component, λ, along the vector connecting the two states (Eq. 1).  λ = 0 refers to the open state configuration, and λ=1 refers to the locked state. For each sampled structure, the value represents the specific state of the structure.

$$\lambda =\frac{\left|v\left(x,y,z\right)-v1\left(x,y,z\right)\right|}{\left|v2\left(x,y,z\right)-v1\left(x,y,z\right)\right|}$$ (1)

λ is the value of the principal component; v  is the position of the sampled structure, v1 and v2 are the positions of the reference structures 1 (the open state) and 2 (the locked state), respectively.

The matrix of distance fluctuation**.** To monitor the structural dynamics and plasticity of the protein, we calculated the matrix of distance fluctuation according to Eq. 2:

$$\tau_{ij}={\left(d_{ij}-d_{ij}\right)}^{2}$$ (2)

where *d*_ij_ is the time dependent distance between atoms *i* and *j*, and bracket represents the time average. Clearly, *τ*_ij_ is independent of the reference structure.

Binding free energy calculations. The Gromacs tool “g_mmpbsa” (Kumari et al., 2014) was used for calculations. For each system, 101 representative frames were evenly extracted from the 100 ns MD trajectory in the production phase. The MM/PBSA method calculates the binding free energy of the protein with ligand in solvent according with Eq. 3:

$$\Delta G_{binding}=G_{complex}-\left(G_{protein}+G_{ligand}\right)\;$$ (3)

$G_{complex}$, $G_{protein}$, and $G_{ligand}$ are total free energies of the protein-ligand complex, isolated protein and isolated ligand in solvent, respectively. These terms were calculated according to Eq. 4, individually:

$$G_{x}=\;\langle E_{MM}\rangle -\;TS\;+\;\langle G_{solvation}\rangle$$ (4)

where $G_{x}$ represents the free energy of isolated protein, or isolated ligand, or protein-ligand complex. *T* is the temperature, and *S* represents the entropy in vacuum. $\langle G_{solvation}\rangle$ is the solvation free energy. $\langle E_{MM}\rangle$ is the average molecular mechanics (MM) potential energy in vacuum, including the contributions from bonded and non-bonded interactions determined by the force field parameters (Eq. 5):

$$E_{MM}=E_{bonded}+E_{vdW}+E_{elec}\;$$ (5)

where $E_{bonded}$ is the bonded interaction energies consisting of bond, angle, dihedral and improper interactions. $E_{vdW}$ and $E_{elec}$ are modeled using a Coulomb and Lennard-Jones (LJ) potential function, respectively. $\langle G_{solvation}\rangle$ is the energy required to transfer a solute from vacuum into the solvent, which is expressed as Eq. 6:

$$G_{solvation}=G_{polar}+G_{nonpolar}\;$$ (6)

where, $G_{polar}$ is estimated by solving the Poisson-Boltzmann (PB) equation, which is the electrostatic contribution. $G_{nonpolar}$ is estimated through solvent accessible surface area (SASA) model to get the non-electrostatic contribution. Through g_mmpbsa, $E_{MM}$, $G_{polar}$, and $G_{nonpolar}$ are calculated individually, which means the binding energy could be decomposed on a per residue basis as Eq. 7:

$$\Delta R_{x}^{BE}=\overset{n}{\underset{i=0}{\sum }}\left(A_{i}^{complex}-A_{i}^{free}\right)$$ (7)

$\Delta R_{x}^{BE}$ is the contribution to the binding energy of residue x. $A_{i}^{complex}$ is the energy of the *i*th atom on x residue in bound state and $A_{i}^{free}$ is the energy in the unbound state.

## Results and Discussion

### Morphology of HAp

**FIGURE 1:**
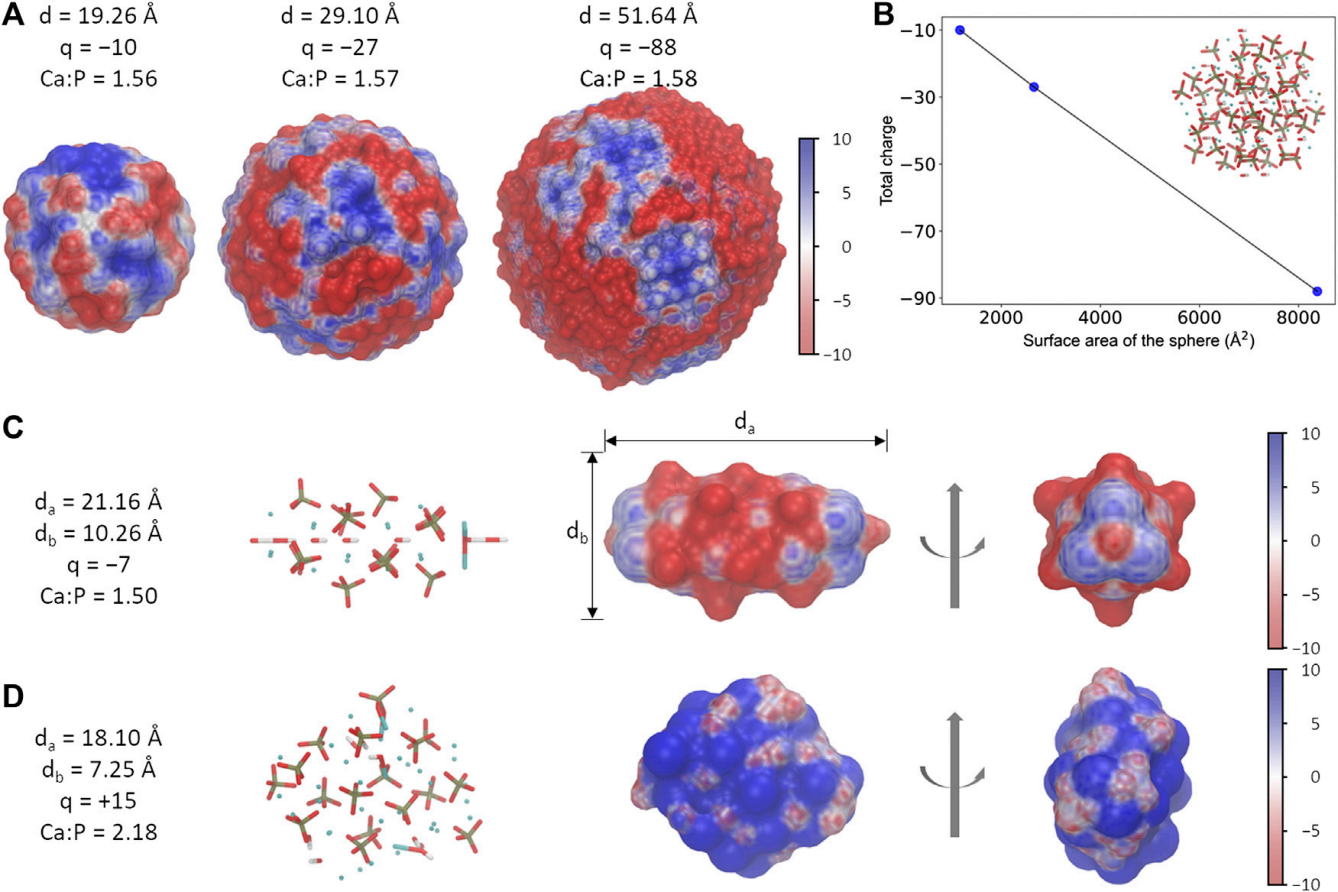
Different morphology of HAp nanoparticles. **(A)** The spherical-shaped HAps with different sizes show slightly different electrostatic potential surfaces. Particles of various sizes are only schematic diagrams and do not correspond to the real scale. **(B)** The ratio between the total charge and the surface area is nearly a constant in HAps. **(C, D)** Information of the needle **(C)** and the rod **(D)** shaped HAps. The unit for electrostatic potential is k_B_T/e.

During the adsorption of proteins on a solid surface, there are several main driving forces, including electrostatic and hydrophobic interactions, as well as the structural rearrangement of the adsorbed proteins (Dee et al., 2003). Due to the highly charged nature of HAps, the electrostatic interaction has been confirmed to strongly affect their affinities toward proteins through charge-charge and charge-dipole interactions (Zhu et al., 2007; Zhu et al., 2010; Chen et al., 2014; Wang et al., 2014). For the HAps with a similar shape (for example, the spherical one) at a given ratio between Ca and P atoms, our calculations show that the difference in their sizes results in certain differences in the electrostatic potential distribution of the surface (Figure 1A). However, the surface charge density of different spheres is close to a constant value, implying a unique electrostatic property at the surface (Figure 1B). Therefore, the HAp-S with the diameter of 1.926 nm was used in the following calculations. On the other hand, changes of the nanoparticles’ geometrical parameters, such as the Ca:P ratio or the aspect ratio, may lead to variable net-charge and the electrostatic potential surface (Supplementary Figure S1), though it was reported that the zeta potentials of HAp nanoparticles of various shape that have been characterized in experiments are all negative (Wu et al., 2019); besides, changes in the size may also lead to a change in the geometric matching of the interface between the two. Therefore, we will focus on the interactions between the fixed-size nanoparticles (Figure 1C,D) and the protein in this work.

Three different morphologies of HAps were examined in this work, which were named as HAp-S (sphere), HAp-R (rod), and HAp-N (needle), respectively. The three systems have distinct electrostatic potential surfaces (EPS) due to different total charges and the charged groups exposed on the surfaces (Figure 1): HAp-S and HAp-N are dominated by negatively charged distributions, while HAp-R is mostly positively charged. More interestingly, the EPSs are not evenly distributed among the three: on HSP-S, the weak positive and strong negative charges are distributed alternately on the surface; on HSP-N, both ends of the needle-like structure are positively charged, whereas the remaining part is mainly negatively charged; the HSP-R features a weak positively charged EPS. Seemingly, the difference in electrostatic potential distribution on HAps' surface indicates their different abilities to interact with the target protein AP2.

### Binding of HAp on AP2-μ2

We then examined the effects of particle morphologies (HAp-S, -R, and -N) on the interactions between endocytic adaptor protein AP2 and HAp nanoparticles. Due to the relatively large size of the AP2 complex and the major role of the μ2 subunit as a cargo-binding region, we focus on the protein structural preservation and alteration of the μ2 subunit (denoted as AP2-μ2) upon the binding of HAp in the following simulations.

**FIGURE 2:**
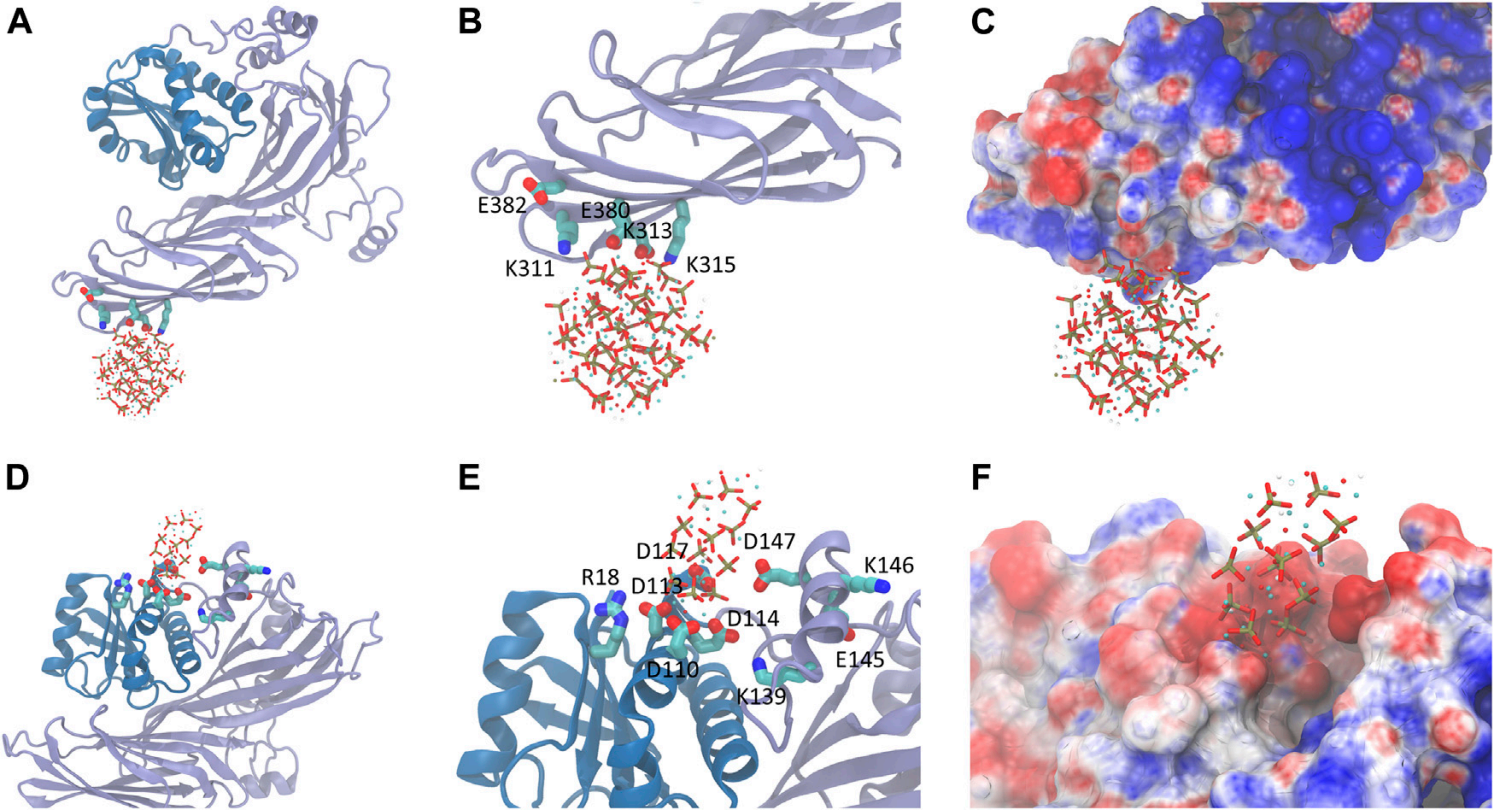
The bound state of HAp-S **(top panel)** or HAp-N **(bottom panel)** on AP2-μ2. **(A)** The binding of HAp-S at the β-sheet-rich C-terminal side of AP2. **(B)** The key residues involved in HAp-S binding. **(C)** The electrostatic potential distribution at the binding site of the HAp-S/AP2-μ2 complex. **(D–F)** Information for the binding of HAp-N. The binding of HAp-N is at the α-helix -rich N-terminal side of AP2.

**FIGURE 3:**
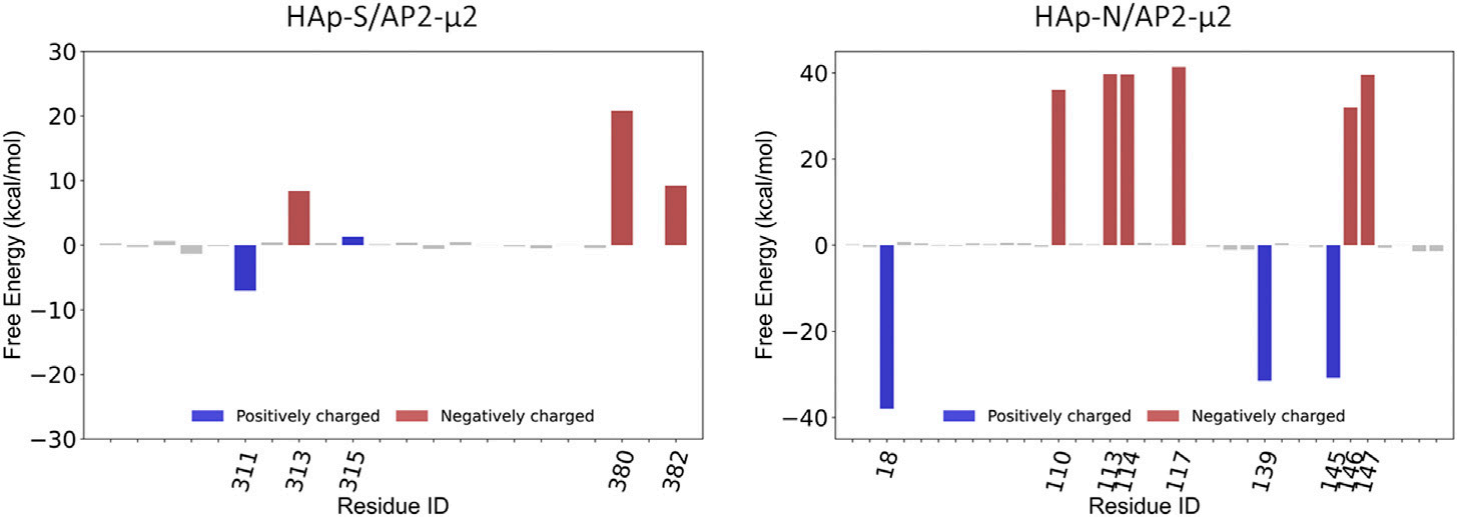
The key residues on AP2-μ2 contributed to the binding of HAp-S **(left panel)** and HAp-N **(right panel)**.

To our best knowledge, no structural information of the complex formed by the HAp and AP2-μ2 is available. Thus, the initial guess about the interface between the two components was derived from rigid-body docking and the matching of electrostatic potential surfaces. Presumably, polar residues K311, E313, K315, E380, and E382 (Figures 2, 3) at the β-sheet-rich C-terminal side of the AP2-μ2 (represented as AP2-μ2-C, residue ID 172-443) mediates the binding, which was designated as binding sites I. It should be noted that this segment is originally deeply buried in the bowl of AP2 core arranged by the α, β2, μ2, and σ2 subunits when it is in a locked state, but is released from the bowl and rotates roughly about its long axis when it is in an open state. Molecular dynamics (MD) simulations show that HAp-S and HAp-N have specific binding sites on AP2-μ2 and consequently cause different conformational changes of the protein.

HAp-S bound to AP2-μ2 tightly at the binding site I throughout the 100 ns simulations. The positively charged residues K311 on the β-sheet-rich C-terminal side of AP2-μ2 retains close interactions with the exposed phosphate groups on the surface of HAp-S. E380 forms stable interactions with an exposed Ca^2+^ on the surface of HAp-S (Figure 2). The HAps well maintain the spherical structure without significant change of its morphology (Supplementary Figure S2), and no release of Ca^2+^ ions was observed within the simulated time scale. Notably, the binding site I is close to the experimentally-identified binding pocket of the μ2-subunit-specific YxxΦ-type-binding motif, which is functionally conserved for AP2 (Kittler et al., 2008).

In contrast, the HAp-N migrated away from the initial binding site I and settled on a pocket (binding site II, Figure 2) formed by α-helices and a number of spatial surrounding amino acids at the α-helix-rich N-terminal region (represented as AP2-μ2-N, residue ID 1-121). One possible reason for the detachment of HAp-N from the binding site I could be attributed to the relatively flat surface at the β-sheet-rich domain. Notably, the binding site II is not fully exposed in the AP2 adaptor complex formed by the α, β2, μ2, and σ2 subunits, but has some overlap with the interface between the μ2 and β2 subunits. In the case of HAp-R, it rapidly detached from AP2-μ2 (5ns after constraint release in the simulations) and remained distant from the protein.

To explore the AP2 conformation and HAp binding sampled in the MD simulations with further details, we clustered the trajectories into six groups by using 1.5 Å root-mean-square deviation (RMSD) as the criterion in each system. Notably, in both AP2-μ2/HAp-S and AP2-μ2/HAp-N complexes, the first three ethnic groups account for more than 85% of all the MD trajectories. Therefore, we calculated the binding free energy with an implicit solvation model, the molecular mechanics/Poisson-Boltzmann surface area (MM/PBSA) method (Kollman et al., 2000; Baker et al., 2001).

Compared with the AP2-μ2/HAp-S complex, the AP2-μ2/HAp-N has a much larger contact area. The total binding free energy indicates that the AP2-μ2 has a much stronger binding affinity with HAp-N than that with HAP-S. Among different interactions, the electrostatic interaction is dominant in both systems, evidenced by several conserved key residues mediating the majority of contributions toward binding.

To further validate the contributions from the charged residues on HAps toward the binding affinity, we also studied the AP2-μ2 with some key residues mutated. We carried out two sets of mutations on the AP2-μ2/HAp-X (X = N or S) complex (Table 1): in the first set, only a single mutation on AP2-μ2 was made, where the residue contributing most to the binding was replaced with a neutral residue alanine. The resulting mutants are AP2-μ2-D117A/HAp-N and AP2-μ2-E380A/HAp-S; in the second set, simultaneous mutations at four important sites toward binding were made. The resulting mutants are AP2-μ2-E110A-D113A-Q143A-E147A/HAp-N and AP2-μ2-R305A-K311A-K315A-E380A/HAp-S. Our data show that mutation of the key residue(s) dramatically reduces the binding between the AP2-μ2 and HAp-X, further illustrating the electrostatic interaction nature between the two.

**TABLE 1:** The binding free energies of the AP2-μ2/HAp-X (X = N or S) complex calculated with MM/PBSA (in kcal/mol).

	HAp-N/AP2-μ2			HAp-S/AP2-μ2		
	Wild type	D117A	E110A, D113A, Q143A, E147A	Wild type	E380A	R305A, K311A, K315A, E380A
*E*_vdw_	9.26 ± 5.20	12.35 ± 4.62	15.60 ± 5.56	−3.31 ± 4.39	−1.32 ± 3.70	0.53 ± 3.61
*E*_elec_	−209.75 ± 18.67	−304.22 ± 10.57	−375.56 ± 36.64	−109.95 ± 14.99	744.56 ± 36.84	582.17 ± 20.70
*G*_polar_	−54.22 ± 15.17	211.44 ± 12.45	224.03 ± 27.80	99.48 ± 39.49	88.49 ± 15.39	52.67 ± 25.81
*G*_nonpolar_	−2.49 ± 0.20	−1.66 ± 0.11	−1.29 ± 0.28	−2.00 ± 0.47	−1.50 ± 0.48	−1.16 ± 0.36
*G*_binding_	−257.20 ± 22.54	−82.07 ± 13.15	−137.22 ± 14.42	−15.78 ± 29.13	830.23 ± 37.76	635.64 ± 27.14

Seemingly, HAps with different morphologies have binding specificity toward AP2-μ2. It should be noted that the inherent limitations of MM/PBSA, as in the lack of implicit information about solvent water around the binding site and large fluctuations of conformational entropy (Hou et al., 2011) hinders the precise calculation of the absolute binding energies. However, given the computational efficiency of this method, the estimated relative values of binding provide meaningful information to distinguish the binding of different HAps.

### HAp-Binding Induced Conformational Change of AP2-μ2

**FIGURE 4:**
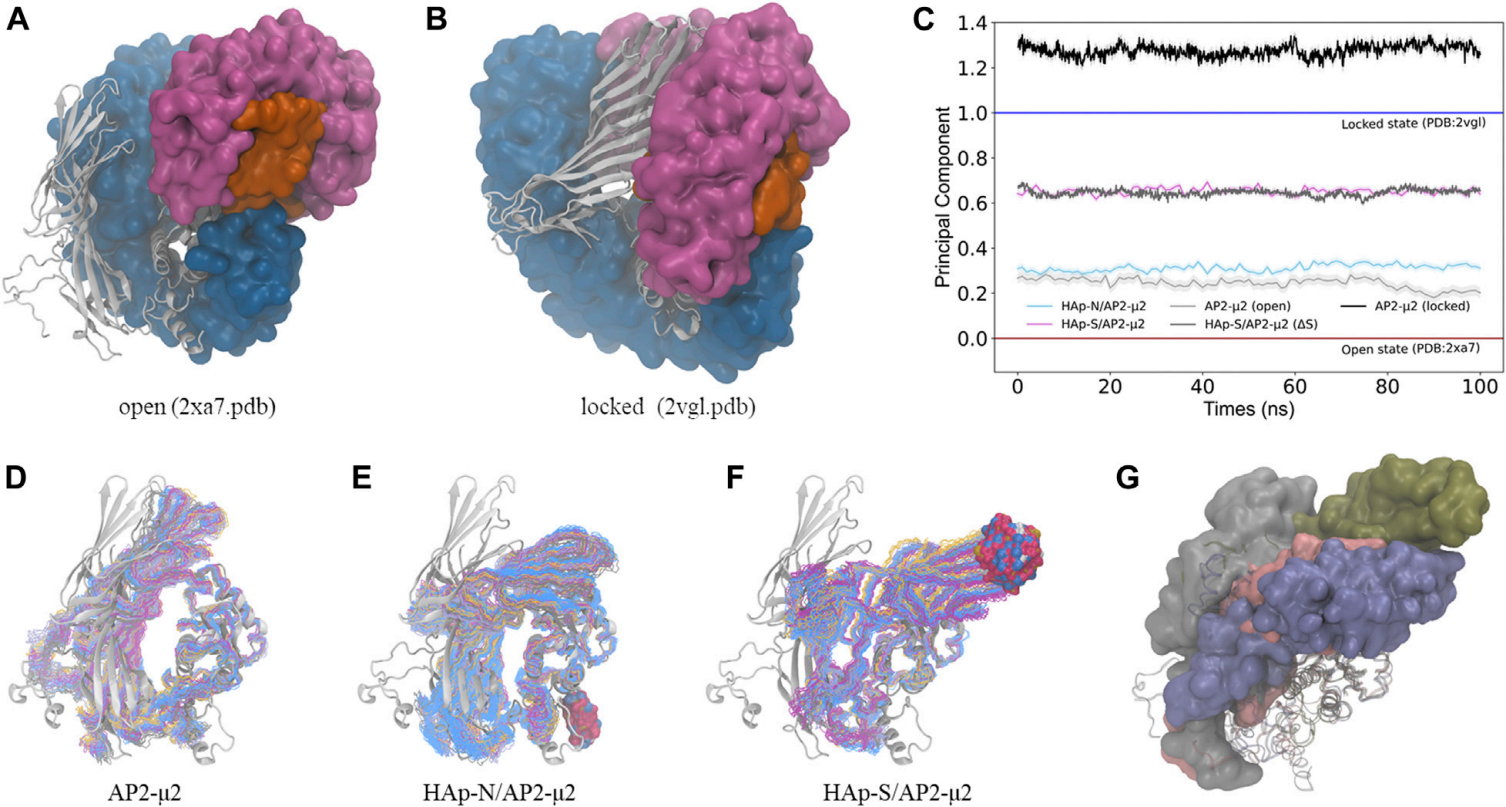
The conformational change of the AP2-μ2 protein upon binding of HAp nanoparticles. **(A, B)** The open **(A)** and the locked **(B)** states of the AP2 protein. The α **(magenta)**, β2 **(blue)**, and σ2 **(orange)** subunits are in the surface mode, and the μ2 subunit **(white)** is in the cartoon mode. **(C)** The calculated principal component of the conformations between the open (λ = 0) and the locked (λ = 1) states of the AP2-μ2 protein sampled with MD simulations. **(D–F)** The sampled conformations (in the line mode) of the AP2-μ2 alone **(D)**, in the bound state with HAp-N **(E)**, or with HAp-S **(F)**, where the AP2-μ2 in the open state is taken from the crystal structure (in the cartoon mode) and shown as the reference. In each ensemble, different colors are used for different clusters of the sampled structures, and the bound HAp particles are shown in the space-filling mode. **(G)** The representative structure of AP2-μ2 in the HAp-N/AP2-μ2 **(pink)**, HAp-S/AP2-μ2 **(blue)** complexes, where the open **(gray)** and locked **(green)** structures are also shown for comparison. The AP2-μ2-C subunit is shown in the surface mode to illustrate the direction of conformational change, and the AP2-μ2-N subunit is shown in the cartoon mode. All the structures are in a view pre-aligned with the AP2-μ2-N subunit. For clarity, the bound HAps are not shown.

To monitor the conformational change of AP2-μ2 upon binding of HAp, we calculated the principal component, λ, of the complex trajectory along a vector connecting the open (Jackson et al., 2010) (PDB entry: 2xa7. pdb, λ = 0) and the locked (Collins et al., 2002) (PDB entry: 2vgl.pdb, λ = 1) states of AP2-μ2 (Figure 4) determined by x-ray crystallography. In the absence of HAp, the conformations of AP2-μ2 generated with MD simulations resemble the open state crystal structure, as indicated by the λ values fluctuating just above 0, showing the stability of open state conformation. Presumably, the small deviation (with the mean value of λ = 0.24) could be attributed to the lack of anchoring subunits α, β2, and σ2 in the present MD simulations.

In contrast, the binding of HAp leads to a certain degree of conformational changes of AP2-μ2. Specifically, AP2-μ2 with both nanoparticles tends to transform to the locked state, as indicated by the value of λ deviating from 0 toward the direction of 1. For the AP2-μ2/HAp-S complex, the average value of λ is ∼0.65, much larger than that of the AP2-μ2/HAp-N complex (∼0.32), demonstrating a more significant conformational change induced by the bound of spherical nanoparticle (HAp-S) than the needle one (HAp-N). In other words, the HAp-N bound AP2-μ2 protein is closer to its open state, while the HPA-S bound one is more comparable to its locked state (Figure 4).

**FIGURE 5:**
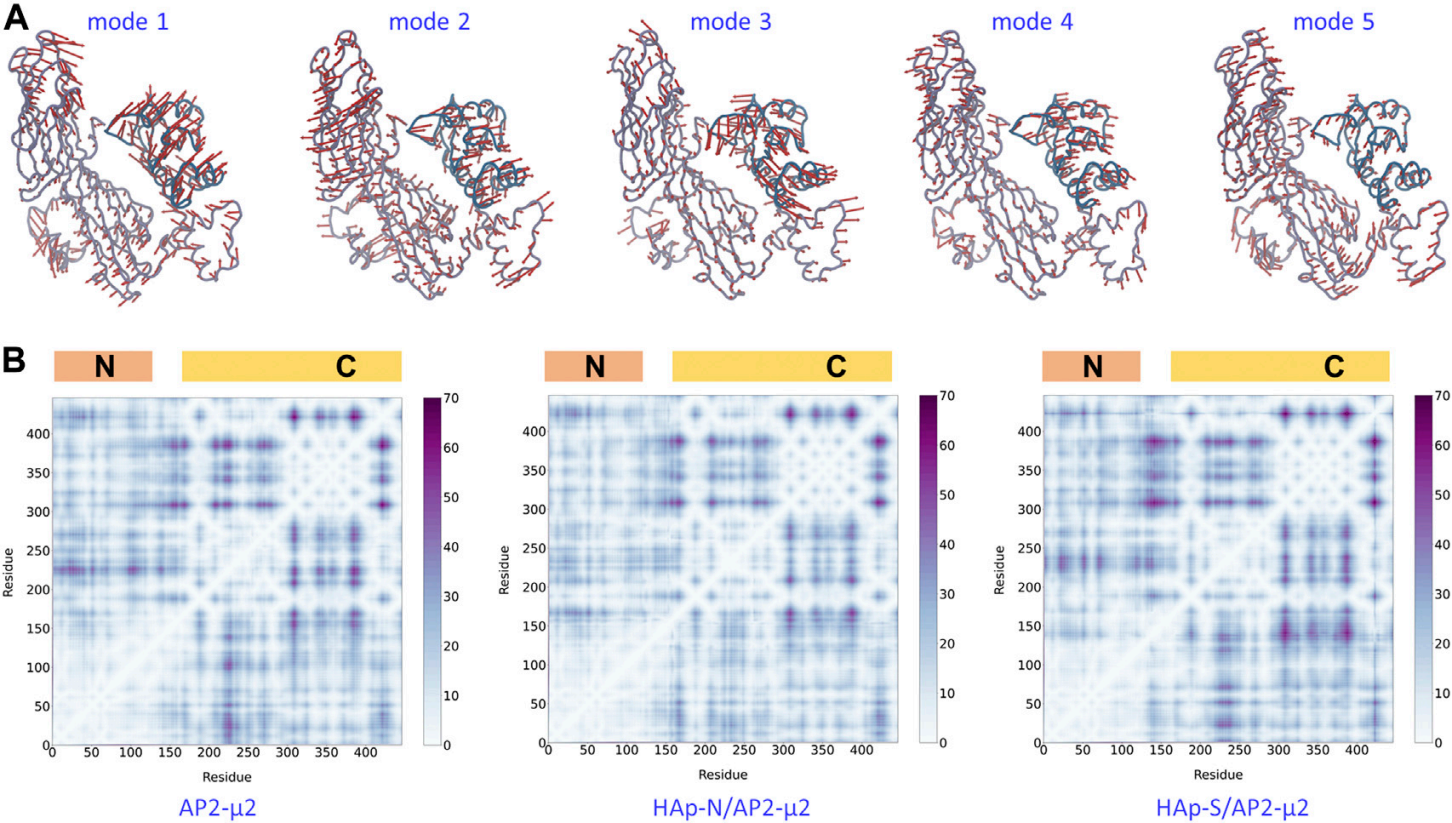
The dynamics and plasticity of AP2-μ2. Top panel: the first five low-frequency modes of collective motion generated with the normal mode analysis on the open state crystal structure. Bottom panel: the matrix of distance fluctuations of the AP2-μ2 protein in the absence **(left panel)** and the presence of HAp-N **(middle panel)** or HAp-S **(right panel)** in the MD simulations. The N- and C-terminal domains are labeled on the top of each panel. The color bar shows the magnitude of fluctuations. Only Cα atoms were used in both calculations.

By comparing the locked and the open state structures (Figure 4), it is clear that the domain rearrangement from the open to the locked state could be described as the rotation of the AP2-μ2-C along its long axis, as well as the approaching of the AP2-μ2-C toward the AP2-μ2-N, as the AP2-μ2-N is relatively fixed in the bowl. We then studied the collective motion of the open state structure of AP2-μ2 with normal mode analysis (Figure 5A), assuming that the system is stabilized by harmonic potentials. The first five low-frequency modes show that relative motions between the C- and N-terminal segments within AP2-μ2 are dominant, indicating the functional role of these intrinsic motions.

The AP2-μ2 experiences different conformational changes in the presence of different HAPs (Figure 4). Presumably, the synergistic effect of the protein plasticity has an impact on the binding affinity of HAp. Therefore, we monitored the matrix of distance fluctuations of the AP2-μ2 protein (Figure 5B). Taking the two domains in the AP2-μ2 as the reference, the binding of HAp-N induces less fluctuations at the N-domain, which is likely to be attributed to its strong binding affinities toward the N-terminal domain (Table 1). Seemingly, the presence of HAp-N locks AP2-μ2 at a certain configuration. In contrast, the binding of HAp-S triggers much larger fluctuations at the C-domain, which is likely to be related to its weak binding affinity. Therefore, HAp-S leads to a more significant conformational change on AP2-μ2 than that of HAp-N (Figure 4).

In the meantime, the binding of the negatively charged HAp partially neutralizes the net charge on AP2-μ2, which is likely to reduce the electrostatic repulsion between the AP2-μ2-N and AP2-μ2-C segments, and therefore facilitate the relative motion between the two. Consequently, HAp-S favors AP2-μ2-C surfaces, while HAp-N is attracted to AP2-μ2-N. Compared to HAp-N, HAp-S possess higher-densities of surface charges and interacts with a relatively exposed protein area (the binding site I). Hence, HAp-S leads to a more significant conformational change on AP2-μ2 than that of HAp-N. In literature, it was reported that the sphere-shaped HAp nanoparticles effectively inhibited the growth of A375 melanoma cells (34.90% viability); in contrast, the rod or needle-like HAp nanoparticles moderately affected the viabilities of melanoma cells (60.43%–74.90%) (Wu et al., 2019). Presumably, HAp-S is likely to facilitate its transportation in the cellular environment by shifting AP2-μ2 to a locked conformation, and therefore resulting in a more profound tumor-suppressive effect.

## Conclusion

In this work, we used molecular dynamics simulations to study the regulation of nanoparticles HAp with the clathrin adaptor AP2. We found that the different morphologies of HAps feature distinct binding affinities toward AP2; the binding of HAps with different morphology leads to structurally and functionally distinct configurations of AP2, which is likely to affect cargo recognition in clathrin-mediated endocytosis profoundly. Our work offers a microscopic explanation for cargo recognition in clathrin-mediated endocytosis and possible mechanisms of designing high-efficiency nano-biomaterials, thus providing a basis for understanding their specificity and potential as intracellular agents.

## Data Availability

The raw data supporting the conclusions of this article will be made available by the authors, without undue reservation.
