# Supplementary material for: The Morphology of Hydroxyapatite Nanoparticles Regulates Cargo Recognition in Clathrin-Mediated Endocytosis
Source: Front Mol Biosci. 2021 Mar 4;8:627015. doi: 10.3389/fmolb.2021.627015 (PMC7969717; doi:10.3389/fmolb.2021.627015)
Supplement: Supplementary file 1 [file table1.docx]

**Supplementary File**

**The morphology of hydroxyapatite nanoparticles regulates cargo recognition in clathrin-mediated endocytosis**

Cheng Zhu^1,2,#,¶^, Xuejie Zhou^1,#^, Ziteng Liu^3^, Hongwei Chen^3^, Hongfeng Wu^4^, Xiao Yang^4^, Xiangdong Zhu^4^, Jing Ma^3,5,^*, Hao Dong^1,5,6,^*****

^1^ Kuang Yaming Honors School, Nanjing University, 210023 Nanjing, China

^2^ Tianjin Key Laboratory of Function and Application of Biological Macromolecular Structures, School of Life Sciences, Tianjin University, 300072 Tianjin, China

^3^ Key Laboratory of Mesoscopic Chemistry of Ministry of Education, Institute of Theoretical and Computational Chemistry, School of Chemistry and Chemical Engineering, Nanjing University, 210023 Nanjing, China

^4^ National Engineering Research Center for Biomaterials, Sichuan University, 610064 Chengdu, China

^5^ Nanxin Pharm. Co., Ltd., 210046 Nanjing, China

^6^ Institute for Brain Sciences, Nanjing University, 210023 Nanjing, China

Email: [donghao@nju.edu.cn](mailto:donghao@nju.edu.cn), [majing@nju.edu.cn](mailto:majing@nju.edu.cn)

^#^: C.Z. and X.J.Z contributed equally to this work

^¶^: C.Z. was a short-term visiting scholar in Kuang Yaming Honors School, Nanjing University


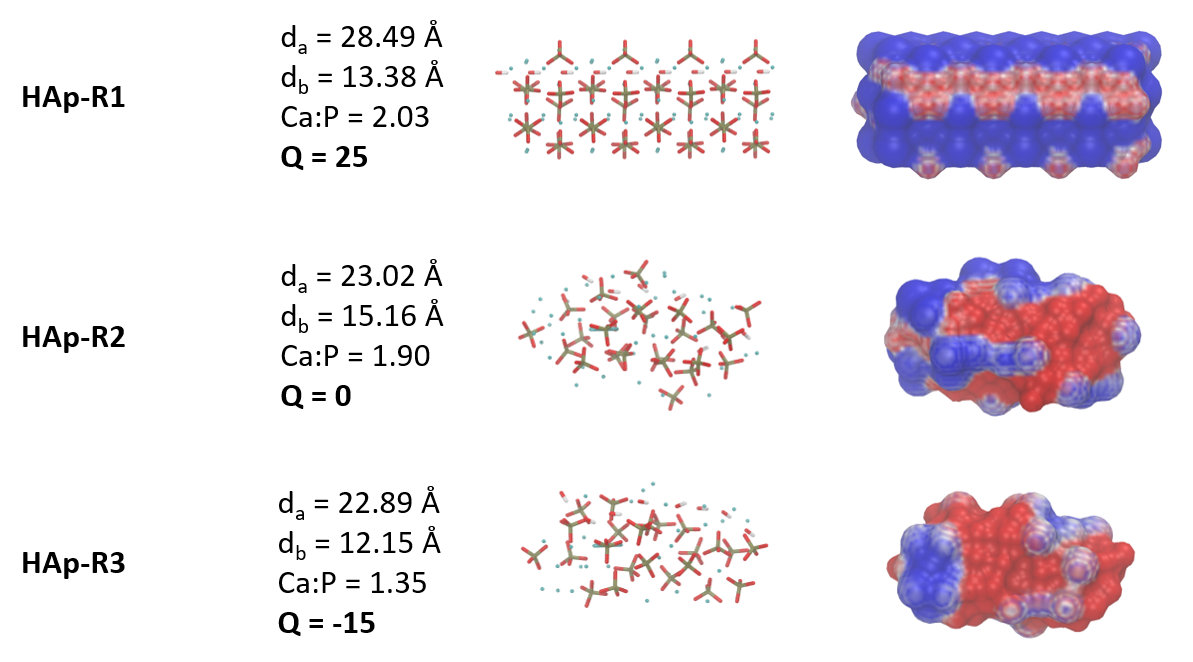


**Figure S1. The rod-like HAp clusters with different geometrical parameters show variable net-charges and electrostatic potential surfaces.** They could be positively charged (top panel), neutral (middle panel), or negatively charged (bottom panel).


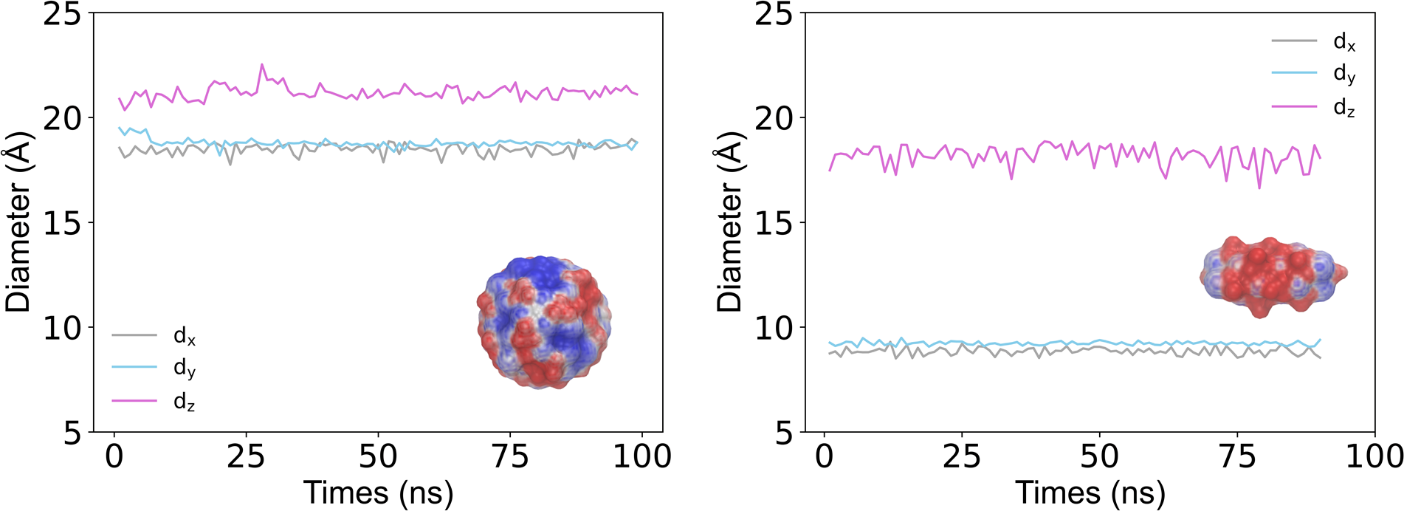


**Figure S2. The structures of the HAp-S (left panel) and HAp-R (right panel) were well maintained in the MD simulations.** The HAp-S and HAp-R structures were extracted from the trajectories. for HAp-R, its three principal axes were aligned to the x-, y- and z-axis, respectively, and the first principal axis was aligned to the z-axis.


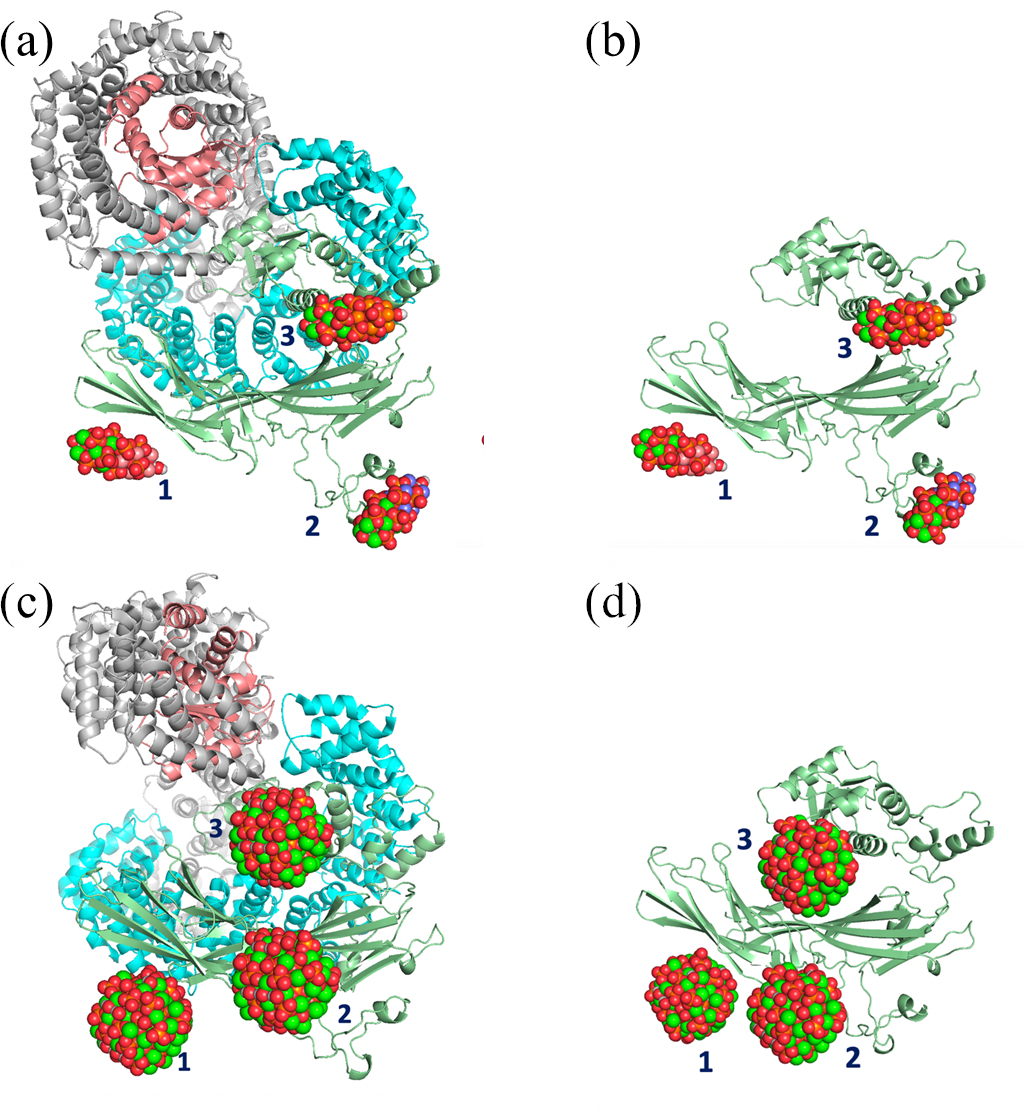


**Figure S3. The three possible binding sites identified by docking on the full-length AP2 protein and the AP2-μ2 only.** (a-b) The three binding sites of HAp-N on the full-length AP2 (a) or the AP2-μ2 only (b). (c-d) The three binding sites of HAp-S on the full-length AP2 (c) or the AP2-μ2 only (d). In each panel, the μ2 domain is shown in cartoon mode (in green), and the rest three domains in the full-length protein (left panel) are shown in gray, pink and cyan. The three possible bound states of HAp are shown in the space-filling mode in each panel.
